# Translation and Cultural Adaptation of an E-Health Program to Promote Positive Mental Health Among Family Caregivers in Portugal

**DOI:** 10.3390/healthcare13010031

**Published:** 2024-12-27

**Authors:** Sandra Carreira, Núria Albacar-Riobóo, Carme Ferré-Grau, Carlos Sequeira, Carmen Andrade, Odete Araújo

**Affiliations:** 1 Health Sciences Research Unit: Nursing (UICISA: E), Nursing School of Coimbra (ESEnfC), 3045-043 Coimbra, Portugal; odete.araujo@ese.uminho.pt; 2 Abel Salazar Institute of Biomedical Sciences (ICBAS), University of Porto, 4050-313 Porto, Portugal; 3 Faculty of Nursing, University of Rovira i Virgili (URV), 43003 Tarragona, Spain; nuria.albacar@urv.cat (N.A.-R.); carme.ferre@urv.cat (C.F.-G.); 4 Nursing School of Porto, 4200-072 Porto, Portugal; carlossequeira@esenf.pt; 5 Center for Health Technology and Services Research & Health Research Network Associated Laboratory (CINTESIS@RISE), Nursing School of Porto (ESEP), 4200-450 Porto, Portugal; carmen.ms.andrade@uac.pt; 6 Department of Nursing, Family and Community Health, School of Health, University of the Azores, 9500-321 Ponta Delgada, Portugal; 7 School of Nursing, University of Minho, 4710-057 Braga, Portugal; 8 Nursing Research Centre, University of Minho, 4710-057 Braga, Portugal

**Keywords:** mental health, caregivers, chronic disease, health program, health literacy

## Abstract

**Introduction:** Caring for a dependent individual, particularly over an extended period, places significant strain on family caregivers, often leading to adverse physical, mental, emotional, social, and economic outcomes for both caregivers and those they care for. Common challenges include anxiety, depression, loneliness, and diminished overall well-being. E-health applications have emerged as effective tools to support family caregivers by promoting positive mental health through online interventions, enhancing problem-solving skills, autonomy, interpersonal relationships, self-control, and a prosocial attitude. **Methods:** This study aimed to translate and culturally adapt the Spanish “Program to Promote Positive Mental Health through the Cuidadoras Crónicos Manual” into the Portuguese context, supporting its implementation as a smartphone application. The process involved translation, back-translation by two native experts, and refinement through a focus group with eight participants. The study adhered to the Consolidated Criteria for Reporting Qualitative Research (COREQ) guidelines. **Results:** The translation and back-translation processes identified several adjustments, which informed discussions in the focus group. Three key themes emerged: (i) conceptual and semantic equivalence, (ii) optimisation of content, and (iii) relevance and timeliness of the manual. **Conclusions:** The Spanish manual for promoting positive mental health among family caregivers was successfully translated and culturally adapted into European Portuguese. Validated through expert input, this marks the first version of the manual tailored to Portuguese caregivers, using Positive Mental Health models to support caregivers of individuals with chronic conditions.

## 1. Introduction

Many European nations, along with regions across the globe, are preparing to address the challenges posed by an ageing population in the coming years. Over the past 50 years, the population aged 65 and older has doubled, increasing from fewer than one million in 1972 to over two million by 2022 [1,2]. Ageing is often associated with the onset of chronic illnesses, many of which are debilitating and limit daily activities. Even if the global prevalence of disabilities and diseases remains steady, the rising number of older adults alone is expected to place demands on healthcare systems that far exceed their current capacities [3].

Informal and unpaid caregiving is crucial in long-term care services in countries like Portugal, where nearly 12.5% of the population was identified as caregivers. Portugal has the highest percentage of co-resident caregivers over 50 [4]. Many individuals care for a spouse, family member, friend, or neighbor who needs assistance with household tasks or personal care. However, this role can be incredibly demanding, frequently leading to physical strain, fatigue, and stress [5]. Family caregivers are often required to provide complex, continuous, and intensive care, demanding specialized knowledge, skills, and significant physical effort. This responsibility frequently necessitates personal and professional sacrifices [6].

Being responsible for caregiving demands a sense of responsibility and the ability to handle various tasks and efforts that can exceed the family caregiver’s capabilities, significantly altering their daily life and priorities. Family caregivers can reach a point where they forget to “take care of themselves, becoming more vulnerable to emotional, physical, and psychological strain” [7]. There is a consensus that providing care for a dependent person, especially for a prolonged period, involves a significant burden with consequences for their health. The burden of juggling numerous tasks and responsibilities can lead to anxiety, depression, panic, and a profound sense of loneliness, which end up having a physical, mental, emotional, social, and economic impact on the lives of both the family caregiver and the person being cared for [8,9]. Evidence suggests that family caregivers show higher levels of psychiatric morbidity than the rest of the population, related to the little attention they pay to their mental health [10]. The degree of exhaustion and the impact on the family caregiver’s health depends on their coping strategies and adaptation to the situation [11].

The negative effects of caregiving are likely to increase due to the rising prevalence of neurocognitive diseases among older individuals, as well as the societal challenges imposed on caregivers of those with chronic illnesses. Future challenges are not vastly different from the current ones and are related to difficulties in managing one’s own life alongside the caregiving role and handling emotions in many situations associated with role reversal and clinical manifestations of the disease [2].

Future challenges should focus on a salutogenic perspective of ageing and informal caregiving. Developing strategies to promote mental health among family caregivers represents a shift from a less remedial paradigm to a more anticipatory approach based on structured interventions [8,10] and the use of application-based solutions, the implementation of which has been accelerated after COVID-19 [11].

The understanding and application of these strategies are influenced by the Mental Health Literacy (MHL) level. A higher level of MHL corresponds to greater knowledge and an enhanced ability to effectively address the challenges and difficulties associated with caring for a dependent individual. Those with higher levels of Positive Mental Health Literacy (PMHL) are more committed to their health and tend to be involved in finding better resources in the family and community through complementary care structures. Health professionals, particularly nurses, must integrate into their intervention how individual, social, and contextual factors influence PMHL, which can improve/potentiate motivation and competence in accessing, understanding, evaluating, and applying knowledge. This increase in PMHL will positively impact Positive Mental Health (PMH) by enhancing problem-solving skills, autonomy, personal satisfaction, interpersonal relationships, and self-control and fostering a prosocial attitude [8]. Therefore, empowering the family caregiver involves preparing them with the skills to mobilize, mobilize technical and scientific knowledge, and make them capable of making the best decisions about their physical and mental health, which, in turn, will provide better care with less physical and mental strain [8,12]. A context with new health needs is proposed, which includes, on the one hand, the transformation of the healthcare system and, on the other, a shift towards more comprehensive care that focuses on the individual and the value of life expectancy in good health [13].

The WHO [14] defines the use of information and communication technologies (such as the internet or mobile applications) for health purposes as e-health. This reflects the transformation of traditional healthcare models, driven by the increasing trend of internet usage. Existing interventions have played a significant role in supporting families caring for dependent individuals, and this support can be further enhanced by leveraging technological innovations. The evidence also suggests that technological resources do not replace the family caregiver in their activities as such, but those can be an excellent resource to promote their mental health and develop strategies, based on mental health literacy, to first take care of their health and, secondly, to promote quality of increasingly complex and prolonged care [13]. Ferré-Grau and his team have developed intervention programs that utilize problem-solving techniques to prevent anxiety and depression [13].

In their systematic review, Lorca-Cabrera et al. [6,15] highlighted a significant gap in research regarding the evaluation of applications designed to enhance caregivers’ health and well-being. The Ferré-Grau et al. [15] randomized controlled trial showed the effectiveness of a digital intervention program in promoting positive mental health among nonprofessional caregivers and evaluated the usability and satisfaction of the app-based intervention program. The results demonstrated that implementing an app-based intervention program for caregivers significantly enhanced their positive mental health. Results showed that the program produced a larger effect on the caregivers’ lives after 3 months of the intervention, which suggests that it is an effective long-term program.

Therefore, it is essential to implement effective online educational tools and programs to support family caregivers of older adults, with or without dementia. Building on this evidence, the present study aims to address the identified needs. Drawing from a previous project that developed an app for caregivers, this study presents a protocol for developing and evaluating a smartphone app-based intervention designed to promote the positive mental health of family caregivers.

The evidence suggests that technological resources do not replace the family caregiver in their activities as such, but those can be an excellent resource to promote their mental health and develop strategies, based on mental health literacy, to first take care of their health and, secondly, to promote quality of increasingly complex and prolonged care [16]. In this sense, more programs are needed to support this group of people, especially those that bring health professionals closer to family caregivers and, at the same time, take care of their mental health. Some of the available evidence highlights the advantages and challenges for the decade 2020–2030, namely ageing in place and using digital solutions through e-health interventions [17].

However, such interventions must be carefully adapted to their target population’s cultural and linguistic context to ensure effectiveness. This study builds on prior work in Spain that developed the “Cuidadoras Crónicos” program, a smartphone app-based intervention designed to promote PMH in caregivers of older people. The Spanish program demonstrated the potential to enhance family caregivers’ biopsychosocial health through structured online interventions.

This study aims to translate, culturally adapt, and validate the program for the Portuguese context, addressing the specific needs of Portuguese family caregivers. By doing so, it not only fills a critical gap in caregiver support tools in Portugal but also provides a model for adapting evidence-based interventions across cultural settings. The validation of this program represents a key step in developing reliable, impactful resources that improve caregiver well-being and promote sustainable caregiving practices.

## 2. Methods

### 2.1. Aim

This study aimed to translate and culturally adapt the Spanish version of the “Program to promote positive mental health through the ‘Cuidadoras Crónicos’ App” into a Portuguese context. The research question focused on how feasible and culturally appropriate the individual items of the Spanish program manual are when adapted for Portuguese focus group participants and how they contribute to supporting the positive mental health of family caregivers.

### 2.2. Design

This study is a qualitative, descriptive, and exploratory design based, firstly, on a translation and back-translation approach and, secondly, on focus group interviews [18].

### 2.3. Procedures

The process of translation and back-translation followed the methodological assumptions by Frey (2018) [19]. The first step preceding this stage was requesting authorization for the manual from the team that developed the original program in the Spanish context. After obtaining authorization through a written agreement, we proceeded to the second stage, which involved translating the manual from the original language (Spanish) to European Portuguese. This stage was carried out by two bilingual nurses whose native language is Portuguese and unfamiliar with the manual and the study objectives. Next, the Portuguese version was back-translated into the original language by two translators whose native language is Spanish, aiming to validate accuracy with the original Spanish version.

After this phase and considering the qualitative nature of this study, we opted to apply a focus group technique to encourage participants to explore and clarify perceptions and perspectives about the main topic of the research study based on collective and group interaction [18]. In addition, this study respected the Consolidated Criteria for Reporting Qualitative Research (COREQ) [20].

The planning phase of this study began with the definitions of the general objectives and the homogeneity of the focus group, the number of members, and the intentional non-probabilistic sampling techniques [21]. The session aimed primarily to assess the agreement and adequacy of concepts in the Portuguese context. Subsequently, the session was developed with general questions outlined, followed by progressively more specific ones [22].

### 2.4. Participants

Potential participants were approached via email, and an invitation was sent two weeks before the focus group session. The email included information on the study’s nature, objectives, participation rules, lack of financial compensation, and expected session duration. The Spanish and Portuguese versions of the manual were also shared with the participants before the session to allow them time for review.

For all participants aged over 18 years old, to be eligible for participation, individuals had to meet the following criteria: (1) be a nurse with clinical experience, (2) be a specialist nurse, and (3) have expertise in the field of mental health and/or family caregiving. The research team initially invited 12 nurses who met these criteria. However, eight nurses ultimately participated due to scheduling conflicts, resulting in a 33% refusal rate. These refusals were anticipated, and a 25% over-recruitment strategy was applied to ensure the focus group met the required size of 5 to 8 participants, as recommended by Krueger and Casey [18].

According to the authors mentioned previously, the focus group technique should include 4 to 12 participants, although it is considered ideal to comprise 5 to 8 participants. Since it is not certain that all invited individuals will participate, it is recommended to have an over-recruitment of at least 25% to avoid potential vacancies that may compromise the minimum number of participants in the focus group [23].

Two researchers conducted data collection, both experienced in qualitative research, mental health studies, and informal care. Their qualifications ensured they were well-equipped to facilitate the focus group discussions and analyze the data effectively. The focus group session lasted approximately 90 min and was held online via the ZOOM platform. This allowed participants to join remotely, ensuring greater flexibility and participation. All sessions were audio-recorded, and additional notes were taken to ensure comprehensive data collection. The confidentiality of all participants was maintained, and informed consent was obtained before the session began [21].

The study participants had varied clinical backgrounds and expertise in mental health and caregiving, ensuring a broad range of perspectives were represented. While the session was not piloted before the main study, a pre-session briefing was provided to ensure all participants understood the focus group’s objectives and structure.

The study did not include repeat interviews. The focus group session was designed as the primary data collection method. It provided valuable insights into cultural adaptation, but we acknowledge that data saturation may not have been fully achieved. This limitation could affect the comprehensiveness of the findings, as some perspectives or experiences may not have been fully captured.

### 2.5. Data Collection

In this study and in addition to the translation and back-translation process of the manual to Portuguese, which took place between June 2023 and February 2024, a focus group session was conducted on 2 May 2024, in European Portuguese as it was the native language of the researchers and the participants who voluntarily agreed to join the study. The research team, specifically the principal investigator, received prior training in conducting the focus group session. Similarly, data collection procedures were agreed on, a script for conducting the focus session was developed, and pre-session training was conducted three days earlier (including only the research team members) to agree on procedures, including the duration of sessions and the control of any unforeseen circumstances. Regarding the questions script, the following steps were followed: (i) welcome and thanks to all participants; team introduction; (ii) presentation of the study and its objectives; (iii) clarification of the roles of the researchers involved: the principal investigator would moderate and conduct the session together with two researchers who would be present as observers, take notes during each session, and manage session recordings; (iv) presentation of some rules and requests, including participants’ consent to record the session. The participants, who were reassured about the compliance with all ethical assumptions inherent in a research study involving humans, were asked not to interrupt the session and to express their opinions as the moderator gave them the floor; likewise, they were informed that they could withdraw from the session at any time without prejudice; (v) no participant would have to pay to participate in the session, nor would they receive any compensation for participating in the research.

Participants were requested to respond to certain questions, which were organized into four main topics/themes:

1. Based on the original version and considering the translation/adaptation of the manual, do you consider it to be in line with the original version?
2. What are the main linguistic/semantic aspects of the program/manual that you consider necessary to change to better meet the needs of Portuguese family caregivers? In other words, is the language of the program understandable? Is it appropriate for Portuguese family caregivers?
3. In your perspective, does this program address the aspects of positive mental health of family caregivers, considering the original model of promoting positive mental health?
4. Considering the program was developed approximately a decade ago, do you think its content is still current? What changes would you suggest?

The meeting lasted 90 min, which respected the international recommendations for conducting a focus group (less than 150 min) [18]. The session recording was transcribed using Transcribe of Microsoft Word (version 365). It included codes to identify all participants, ensuring their anonymity (P1—Participant 1; P2—Participant 2… P8, Participant 8).

### 2.6. Data Analysis

After the focus group session, all data were analyzed systematically and rigorously [18]. One researcher transcribed the session’s content into the native language (European Portuguese). Subsequently, and after full transcription, two researchers carried out a thematic analysis according to the assumptions of Braun [24,25] and organized it into six steps: “(i) Familiarizing yourself with your data: transcribing data (if necessary), reading and re-reading the data, noting down initial ideas. (ii) Generating initial codes: coding interesting features of the data systematically across the entire data set, collating data relevant to each code. (iii) Searching for themes: collating codes into potential themes, gathering all data relevant to each potential theme. (iv) Reviewing themes: checking if the themes work in relation to the coded extracts (Level 1) and the entire data set (Level 2), generating a thematic ‘map’ of the analysis. (v) Defining and naming themes: ongoing analysis to refine the specifics of each theme and the overall story the analysis tells, generating clear definitions and names for each theme. (vi) Producing the report: the final opportunity for analysis. Selection of vivid, compelling extract examples, final analysis of selected extracts, ensuring the analysis addresses the research question and literature, producing a scholarly report of the analysis” [25].

Data analysis was initially carried out as a team using Microsoft 365. Within this package, the transcription functionality of Microsoft Word was used. This process aims to familiarize the team with the findings obtained from the focus group sessions and ensure consistency in the naming of codes. The initial coding and theme development were carried out in a way that facilitated a reflective and in-depth thematic analysis [24]. This method fosters reflection to deepen understanding and strengthen the connection with the themes. The group subsequently revisited the themes developed in the initial sessions for further classification, refinement, and alignment with the themes and codes identified by the research tea.

### 2.7. Ethical Considerations

Ethical approval for this study was obtained from the Comissão de Ética da Sociedade Portuguesa de Enfermagem de Saúde Mental (Reference CEASPESM_4/2023). Before data collection, all participants were thoroughly informed about the study’s objectives, procedures, and their rights as participants, including the voluntary nature of their participation and their ability to withdraw at any time without penalty. Written informed consent was obtained from all eligible participants.

The study adhered strictly to the ethical principles outlined in the Declaration of Helsinki (59th Amendment) and the Oviedo Convention, ensuring the protection of human rights and dignity during the research process. Participants’ confidentiality and anonymity were rigorously maintained, and all data were securely stored and used solely for research purposes.

These measures reflect our commitment to conducting ethically sound research that respects the autonomy and well-being of participants.

## 3. Results

After translating the manual, we proceeded with the back-translation of the manual with the help of two experts who are native speakers of the manual’s original language. The results from the first expert showed 20 semantic changes, and from the second expert, 6 semantic changes. Examples of some expressions can be analyzed in European Portuguese (Table 1). After back-translation, we compared the back-translated version with the original text to identify differences or deviations in the mean. Then, we evaluated the semantic equivalence between the two versions, ensuring that the original text’s meaning, context, and intent have been preserved. In the final review, we made the necessary corrections to the translation based on the discussions, aiming to ensure that the final text is faithful to the original and comprehensible in the new language.

The results from the socio-demographic characterization focus group are presented in Table 2. The sample consisted of eight female nurses with expertise in mental health and informal care. Seven of them had been nurses for more than ten years, and one had been working for over 30 years. All participants had post-graduation education; three were holders of a master’s degree, and five held a doctoral degree (Table 2).

Three themes emerged from the question in the focus group (Table 3).

### 3.1. Theme 1—Conceptual and Semantic Equivalence

All participants stated there was consensus on translating the Spanish language manual into Portuguese. However, in response to the second question of the semi-structured interview, they pointed out the importance of conceptual and semantic equivalence. They mentioned the importance of standardizing the language and pointed out some conceptual aspects. The first relates to the title: “P3: *But my question that caught my attention was right away the title of the application, chronic caregivers, which doesn’t make sense to me, first, because somewhere in the program, the term ’caregiver’ appears, and in this sense, the title should be caregivers of people with chronic illness*”. P4: “As *we are discussing positive mental health, it would be better to give it a title that is not as burdensome as ’chronic caregivers’. So, perhaps we should try for a lighter, more suitable title here. We are here to promote positive mental health*”.

They also mentioned that some words, despite having an adequate translation, should be adapted to the Portuguese context, such as primary care replaced by primary health care P1: “*Okay, what I’d like to emphasize is that here in Portugal, it’s not primary care. It’s primary health care*”, as P3 reinforces: “*And to talk a bit about the more common language, then also the question of primary care*”. They also suggested using the word caregiver instead of woman caregiver, nurse instead of woman nurse, and person instead of patient, and replacing Genial with good—P1: “*Then I was very, very, very doubtful, why woman caregiver? I understood that there is an explanation for being a woman caregiver, but we must think that maybe here in Portugal, we have a lot of caregivers, too.*” P3: “*I emphasize this issue of carer versus cared for*”. P4: “*(…) also look here for the scouter person client, but also have a term standardization. I disagree that patients and patients are much less, so they’re not all sick, are they? They are, in fact, people who need to be empowered to continue their work*”. P5: “*I think it’s great when we’re talking about a conversation, and I know that in terms of translation, it’s completely correct (…) I would change it to good because when I want to answer the question, how was the conversation, I don’t say genial; I say good*”.

### 3.2. Theme 2—Optimization of Content/Activities

During the discussion, the importance of conceptualizing alternative responses and fewer directive options for family caregivers was emphasized to foster their participation and promote positive mental health—P3: “*I think our caregivers need a more comprehensive and less directive language. I suggest using phrases throughout the application that are not mandatory but are carefully guiding the behavior and actions we want the caregivers to take*”. P5: “*I agree with the previous colleague that the application is somewhat directive. Besides being a bit directive, I also think we could improve it to make it more welcoming*”. P8: “*Looking at the activities, for example, they vary, with some being more directive. We should start or show some care, nurturing the caregiver because this is an application*”.

To better adapt the content and activities to the Portuguese context, participants highlighted the importance of cultural habits, customs, and traditions—P3: “*Adjust it to cultural aspects. For instance, the music suggestions. Everyone will recognize some songs by Mariza that evoke emotions and feelings, giving a Portuguese touch to the cultural adaptation*”. P4: “*Knowing the Portuguese culture, I think we should ensure the application meets the needs of the people*”. P8: “*Regarding the music, I agree with the previous points*”.

The symbolism and imagery in the manual were also focused on. Many participants felt the current imagery was reductive and did not appeal to positive mental health. They suggested that a plant, tree, or starfish could be used instead—P1: “*I was wondering why an onion? It reminds me of women in the kitchen. For example, a starfish or a plant that grows from a seed, representing mental health growth, would be interesting*”. P3: “*I have seen many positive mental health promotion programs that use flowers*”. P4: “*I thought it was a turnip, not an onion. But I agree that a plant or tree, which grows and takes root, would be better*”.

Participants also suggested that some dynamic activities, such as creating sharing groups or thinkers’ groups or allowing caregivers to write and share their thoughts, could be used—P3: “*Research has shown the benefits of caregiver groups*”. P4: “*We could include a feature for caregivers to write something related to the activity, reflecting on their experiences*”. P5: “*Groups where caregivers share and reflect on their challenges could be very helpful*”.

They also highlighted the importance of positive reinforcement for caregivers at the end of each activity—P1: “Expressions that invite continuation, like ‘keep going, you’re on the right track’, instead of just ‘congratulations’ or ‘well done’”. P3: “Highlight the excellent care they provide to their family members, enhancing their personal growth and satisfaction”. P4: “Encourage caregivers to reflect on their positive experiences and personal growth”. P8: “Ensure caregivers understand the overall benefits of the activities, motivating them to continue”.

Promoting self-management of emotions was also considered to be crucial in getting caregivers to join the program and cultivate self-awareness. P4: “*Exercises for recognizing and managing emotions should be included, as they are important for self-awareness and emotional regulation*”. P5: “*Self-knowledge and emotion management should be emphasized to enhance the caregivers’ overall experience with the application*”.

Finally, the importance of addressing the post-caregiving period was mentioned, as this is a significant phase for positive mental health—P5: “*We should consider the post-caregiving period when caregivers need support in adapting to a new phase of life*”. P8: “*It’s very important to address the post-caregiving phase*”.

### 3.3. Theme 3—Relevance and Timeline of the Program/Manual

In the final question of the semi-structured interview, participants discussed the relevance and current applicability of the program. Despite being developed a decade ago, the program remains relevant as it is aligned with the digital health literacy of caregivers and promotes the adoption of new technologies. P4: “*The program will be delivered through an application addressing digital literacy among caregivers*”. P1: “*Our elderly caregivers are quite advanced in digital literacy, especially since they started using WhatsApp during the COVID-19 pandemic*”. P8: “*Many caregivers over 70, some even in their 80s, already use smartphones and social media, which is crucial for digital literacy*”.

All participants agreed on the relevance of the theme and its ease of use, highlighting its value in promoting positive mental health for caregivers—P3: “*I think the program meets the six principles of positive mental health promotion*”. P5: “*It aligns with the main principles of positive mental health, though we could improve the engagement aspect*”. P8: “*The program adheres to the positive mental health model, though it may need some updates after ten years*”. P7: “*It fulfils the positive mental health promotion criteria*”.

## 4. Discussion

This study aimed to translate and culturally adapt the Spanish version of the “Program to promote positive mental health through the ‘Cuidadoras Crónicos’ App” into the Portuguese context. It describes all stages of translation and cultural adaptation, using native language experts to translate the manual and two Spanish natives for the retroversion, aiming to validate agreement with the original Spanish version. Both native Spanish researchers have suggested optimizing the translation of the manual from Spanish to Portuguese. The first researcher suggested 34 changes to words and semantics, and the second made 28 changes. After that, the manual was given to mental health and informal care experts for the cultural adaptation of the manual (focus group).

The experts emphasize the importance of ensuring conceptual and semantic equivalence. Adapting instruments, manuals, or programs is a complex process that requires specialized resources with scientific and technical expertise in both the subject matter and the native language of the program, instrument, or manual being validated [26]. Often, translation is mistakenly perceived as merely converting content from the original language to the target language. However, translation is an act of performance and language use that can be seen as a process of recontextualization. This process not only reshapes the language but also removes it from its original context and places it into a new one, where different values are assigned to communicative conventions, genres, and reader expectations [27]. As highlighted in the study, this is not just about translation; it involves a crucial phase of language standardization, activity updating, and linguistic reformulation. Another important issue highlighted was the importance of optimizing content and activities to ensure the integration of cultural habits, customs, and traditions. This includes the suitability of symbolism and imagery and the enhancement of activities.

Equally important was the need to optimize content and activities to ensure integration. One of the biggest challenges in this type of work/research is related to the requirements of the translation process and translating the meaning of the content rather than word-for-word. The initial objective of translation is to preserve the same interpretation of meaning across different cultures, requiring an explanation to the translators involved in the research. From a linguistic point of view, it is necessary to understand the grammar and semantics of the original content, that is, the country’s cultural context and language of origin. The combination of requirements and specificities justified the involvement of various actors. Professionals with deep knowledge (native) in the original language (Spanish) and professionals, namely nurse experts in mental health nursing and in the field of aging and care for older people with chronic diseases, were involved. The panel of experts that comprised the focus group comprised professionals from clinical practice contexts in hospital and community settings and university faculty members with expertise in the field.

According to Hall (2016) [28], for the cross-cultural adaptation of instruments/manuals to be successful, it is necessary to consider all elements to be translated and adapted to the context, including the title, introductory text, target audience, and manual usage procedures. From the perspective of these authors, the adaptation process comprises six steps: (1) preparation, (2) translation from the original language to the target language (forward translation), (3) translation from the target language back to the original language (backward translation), (4) committee review, (5) field testing, and (6) translation review and finalization.

From our perspective, the translated version of the manual into Portuguese faithfully preserved the contents of the original Spanish manual. As expected, adjustments had to be made despite the proximity between the two countries (Portugal and Spain). Specifically, semantic and conceptual changes were made, and themes were added to preserve the relevance and timeliness of the study object.

Evidence strongly supports the superior effectiveness of culturally adapted interventions compared to non-adapted ones in improving clinical outcomes. Cultural adaptation ensures that interventions are linguistically accurate, contextually, and culturally relevant, increasing their acceptability and impact. A notable example is the adaptation of iSupport for multiple contexts in the world [29], which is anticipated to provide significant support to family caregivers of dementia patients in Portugal by aligning the intervention with their cultural values, practices, and needs [30].

Additionally, resources provided by caregivers, Portuguese habits, customs, and traditions were included, as well as changes to the imagery. The original imagery, depicting the development of onion, was deemed by experts to represent the evolution or demands of informal caregiving inadequately and to misalign with the dimensions of the multidimensional model of positive mental health. Creating the logo also enables recognition of the program, making it more familiar to the user: the family caregiver. This, in turn, facilitates reaching all family caregivers and the public, effectively conveying essential information for promoting positive mental health. Choosing Starfish as the logo for a program aimed at enhancing the positive mental health of family caregivers carries profound and meaningful symbolism. Firstly, the starfish is known for its ability to regenerate lost limbs, symbolizing resilience, overcoming challenges, and renewal. These attributes reflect the qualities the program seeks to foster in family caregivers, helping them recover from emotional challenges and strengthen their mental health. As an element of nature, the starfish represents simplicity, balance, and harmony—essential values for maintaining good mental health and coping with the demands of caregiving. Another reason is that the symmetrical structure of the starfish can symbolize stability and balance, traits the program may aim to cultivate in caregivers to help them better manage their responsibilities and promote self-care. The starfish is a friendly, easily recognizable, and accessible image that evokes inspiration, calm, and hope, reinforcing the program’s positive message and its focus on emotional well-being. This makes the logo more welcoming and universal, fostering identification and connection with the program’s target audience.

We only reached the final version after including a panel of experts who made substantive contributions to cultural adaptation regarding culturally accepted concepts and expressions and made it possible to incorporate current themes that respond to current societal challenges. Specifically, it was only with the know-how and expertise of those experts that it was possible to include themes centered on post-care (e.g., after the death of the cared person), positive reinforcement of the caregiver’s role, and self-management of emotions.

The third theme that emerged was the relevance and timeliness of the program/manual. This work is part of a multicenter project to evaluate the effectiveness of a positive mental health promotion program for caregivers of people with chronic illnesses. Additionally, it seeks to promote digital literacy, which has become increasingly relevant and crucial in the post-COVID era.

This study corroborates that providing guidance and support to informal caregivers is one of the priority areas identified by the World Health Organization to improve the quality of life of family caregivers and their families [31]. Internet-based interventions are more easily accessible [32,33] and adaptable to family caregivers’ time and geographic constraints [34]. Some reviews suggest that multiple components of digital interventions can reduce the burden and improve the quality of care, and they can be even more beneficial if tailored to the family caregiver’s specific needs and contexts [35,36,37].

This manual, which will support an app that will support family caregivers, is essential for maintaining the well-being of both the caregiver and the care recipient. By implementing coordinated interventions aimed at addressing caregivers’ needs, we can enhance their satisfaction with caregiving and ultimately improve the quality of life for everyone involved. Research highlights the importance of providing adequate support for caregivers to improve their care quality. Given the emphasis on ensuring that family caregivers are adequately supported, it is crucial to understand the types of support they want services and organizations to prioritize [38].

This support can range from educational resources and training to care services and emotional support networks. Overall, prioritizing caregiver support can lead to more effective caregiving practices and better outcomes for all parties involved [8]. The World Health Organization (WHO) has defined a long-term strategy for expanding and using digital health, emphasizing its positive impact on healthcare access and delivery and on the health and well-being of both the population and caregivers [39].

To finalize, this application was developed in Spain and impacts the positive mental health of family caregivers, as confirmed by Ferré Grau [15], with the randomized control trial, which validates the importance of adapting this app to the Portuguese context.

### Strengths and Limitations

This study presents some strengths as well as some limitations that should be reported. The first strength is that this study employed a rigorous methodology for cultural adaptation, including translation, back-translation, and focus group discussions, ensuring the linguistic and cultural relevance of the adapted manual. The second strength is the involvement of focus groups, which allowed for valuable insights from the target population and facilitated adjustments to improve the comprehension and appropriateness of the materials. By emphasising qualitative approaches, the study captured nuanced cultural considerations that quantitative methods alone might overlook, enhancing the contextual sensitivity of the adapted program.

As a limitation, we point out that the focus group session was conducted in a virtual context, which may have reduced participant interaction. However, considering that the experts are from very different institutions that are geographically distant from the original location (University of Minho), we believe that if the meeting had been face-to-face (presential), the participation would have been lower, or it might not have been possible to conduct the focus group session at all. Another limitation is that this study did not include statistical testing for the reliability or validity of the adapted materials, as the primary focus was on cultural adaptation rather than psychometric validation. These steps are recommended for future research to ensure the effectiveness and consistency of the adapted program. A further limitation is that while the study focused on cultural adaptation, it did not assess the program’s direct impact on caregivers’ positive mental health, which should be addressed in subsequent research. While the focus groups provided valuable insights into the cultural adaptation process, we acknowledge that data saturation may not have been fully achieved. This limitation could affect the comprehensiveness of the findings, as some perspectives or experiences may not have been fully captured. Future studies should consider including additional focus groups or a larger number of participants to ensure data saturation and enhance the robustness of the findings.

## 5. Conclusions

In this study, the Spanish program “Promoting Positive Mental Health through the ‘Cuidadoras Crónicos’ App” was translated and culturally adapted to the European Portuguese context. This process included validation by experts with recognised experience in mental health, ageing, and informal caregiving. The cultural and linguistic validation ensures the program is tailored to the sociocultural specificities of the target audience, increasing its acceptance and potential impact in promoting positive mental health. However, some limitations must be acknowledged. The virtual format of the focus group sessions, while necessary due to geographical constraints, may have affected participant interaction. Additionally, the study did not include statistical testing for the reliability or validity of the adapted materials, focusing instead on cultural adaptation. Finally, while the insights from focus groups were invaluable, the lack of data saturation could have limited the comprehensiveness of the findings. Future research should address these limitations by conducting psychometric validations, assessing the program’s impact on caregivers’ positive mental health, and including more participants to ensure robust conclusions.

This study provides significant contributions because it is a pioneering work in the Portuguese context. This is the first validated program in Portugal using Positive Mental Health (PMH+) models specifically aimed at family caregivers of older people, addressing an important gap in the adaptation of evidence-based interventions for this specific population. This work establishes a solid methodological and scientific foundation for developing ongoing digital solutions, such as the mobile application (App), which will be an essential tool to support family caregivers.

This study addresses a recognized need in literature for culturally adapted and validated digital interventions for informal caregivers, paving the way for future studies evaluating the effectiveness of such solutions on a larger scale.

Thus, this study represents a crucial initial step toward creating an innovative application and contributes to the applied science of informal caregiving and positive mental health, aligning with global priorities for caregiver support and mental well-being promotion.

## Figures and Tables

**Table 1:** Semantic changes of back translation.

Translation Version	Back Translation Version	Final Translation Version	Expert (E) Code
Assumem	Asumen	Acarretam	E1
oferecer	Ofrecer	Prestar	E1
uma atenção	una atención	Cuidados	E1
Atenção primária	Atención primaria	Cuidados de Saúde Primários	E1
coletivo	Colectivo	Grupo profissional	E1
paciente	Paciente	Utente	E1; E2
condições	Condiciones	Situações	E1
cotidiano	Cotidiano	Quotidiano	E1; E2
seguinte	Siguiente	Feito	E1
tela	tela	Ecrã	E1; E2
pôr	Poner	Colocar	E1
desfrutar	Disfrutar	Aproveitar	E1
a TIVA	la TIVA	TIVA	E1
tónicos	Tónicos	Sentidos	E1
adicionalmente	Adicionalmente	além disso	E1
diante	Delante	Perante	E1
tomar	Tomar	Ter	E1
Enfermeira	Enfermera	Enfermeira/enfermeiro	E1
Médico	Médico	Médica/médico	E1
Cuidadores	Cuidadores	Cuidadores crónicos	E1
óptimo	Óptimo	Ótimo	E2
sentimientos	Sentimientos	Sentimentos	E2
desconectar	Desconectar	Desligar	E2

**Table 2:** Socio-demographic characterisation.

Variables		No.
Sex	Female	8
	Male	0
Age	30–40	1
	40–50	6
	50–60	1
Time working as a nurse	0–10	0
	10–20	7
	20–30	0
	30–40	1
Area of expertise	Mental health	6
	Informal Care	2
Academic Degree	Master	3
	Doctor (PhD)	5

**Table 3:** Themes, categories, and codes.

Theme	Definition	Categories	Codes
Theme 1	Conceptual and semantic equivalence	Standardization of language	Primary care–primary healthcare Title–chronic caregivers Woman Caregiver–caregiver Woman Nurse–nurse Patient–person Great–good
Theme 2	Optimization of content/activities	Updating of activities and linguistic reformulation	Alternative answers and less reductive options for caregivers
		Cultural habits, customs, and traditions	Portuguese music
		Suitability of symbolism/image	Onion–plant/star
		Boosting the activities	Sharing groups/thinkers Writing/sharing something by the caregiver Positive reinforcement of the caregiver’s role Self-management of emotions Post-caregiving period
Theme 3	Relevance and timeline of the program/manual	Digital health literacy	Adoption of new technologies
		Pertinence of the topic/ease of use	Positive mental health

## Data Availability

The datasets used and/or analyzed during the current study are available from the corresponding author upon reasonable request.

## Abbreviations

COREQ—Consolidated Criteria for Reporting Qualitative Research
